# ι-Carrageenan nanocomposites for enhanced stability and oral bioavailability of curcumin

**DOI:** 10.1186/s40824-021-00236-4

**Published:** 2021-10-09

**Authors:** Joo Young Lee, Sanghee Lee, Jang Ho Choi, Kun Na

**Affiliations:** Department of Biotechnology, Department of Biomedical-Chemical Engineering, The Catholic University of Korea, 43 Jibong-ro, Wonmi-gu, Bucheon-si, Gyeonggi-do 14662 Republic of Korea

**Keywords:** Carrageenan, Curcumin, Sulfate group, Intestinal permeability, Stability

## Abstract

**Background:**

Carrageenan (CRN), a polygalactan consisting of 15 to 40% ester sulfate, is used in oral controlled-release technology due to its viscosity and large molecular weight. Curcumin (Cur) is a highly potent antioxidant and anti-inflammatory agent against various diseases, such as tumors, liver disease, rheumatism, and Alzheimer’s disease. Although Cur shows excellent effects in the body, it has major problems, such as poor solubility and low bioavailability in water.

**Method:**

Nanocomposites containing Cur were developed by emulsion technique. Cur@CRN was characterized through the viscosity measurement, size analysis, stability test, and loading efficiency. Antioxidant effects was analyzed with DPPH reagent, and anti-inflammatory effects was analyzed by NFkB/IkBr signaling pathway with wester blot. Cellular interaction was confirmed by flow cytometry and confocal images. Especially, permeability test was demonstrated in MDCK and Caco-2 monolayer cells.

**Results:**

Cur@CRN enhanced stability, antioxidant, and anti-inflammatory effects in vitro, compared with other polymer nanocomposites. Sulfate groups (SO_4_^2−^) in CRN are transported across cell membranes by anion exchangers of the SLC26 gene families. We confirmed Caco-2 cells expressed SLC26A2 receptors interacted with CRN, expect for Tween 80 and hydroxypropyl cellulose. In contrary, other cells did not interact with CRN due to non-expression of SLC26A2 receptors. Based on this, Cur@CRN showed 44-fold better permeability than free Cur in MDCK cell assay.

**Conclusion:**

Enhanced intestinal permeability of Cur can be applied in various health care facilities with significant antioxidant and anti-inflammatory effects compared with nonformulated Cur. Since the CRN composed of nanocomposites has a high molecular weight, high viscosity, and sulfate groups, it will be a platform that can increase the bioavailability of various insoluble drugs as well as Cur.

**Supplementary Information:**

The online version contains supplementary material available at 10.1186/s40824-021-00236-4.

## Background

Carrageenan (CRN), a family of high-molecular-weight sulfated polysaccharides, is obtained from red seaweeds by extraction [1]. It is composed of alternate D-galactose units and 3,6-anhydrogalactose joined by α-1,3- and β-1,4-glycosidic linkages. CRN is separated into various types (such as λ, κ, ι, ε, μ), which are classified by the position and number of sulfate groups as well as the content of 3,6-anhydrogalactose [2]. ι-CRN has an ester sulfate content of approximately 28 to 30% and 3,6-anhydrogalactose content of approximately 25 to 30% [3]. CRN with high molecular weight (above 100 kDa) that is orally administrated can penetrate the mucosal barrier of adult animals via Peyer’s patches [4] and the interaction of sulfate and solute carrier 26 (SLC26) transporters [5]. The application of CRN is being actively studied in oral drug delivery due to its high stability in highly acidic gastrointestinal environments [6, 7].

Orally administered drugs are absorbed systemically from the small intestine by influencing two major superfamilies of membrane transporters (ATP-binding cassette and solute carrier) [8, 9]. In the cell membrane, proteins involved in solute transport into and out of cells are expressed [10–12]. Dietary sulfate is absorbed through the intestinal epithelium by activating the SLC26 family, which is known as a multifunctional anion exchanger and anion channel transporting a broad range of substrates such as sulfate, oxalate, Cl^-^, and HCO_3_^−^ [13]. SLC26 families have been identified with SLC26A1 and SLC26A2 [14, 15]. Although SLC26A1 is located on the basolateral intestine, hepatocytes, and renal proximal tubular cells [16, 17], SLC26A2 is highly expressed in the luminal membrane of colonic crypts as a major site of SO_4_^2−^ absorption [18, 19]. In addition, sulfate transporters and anti-sigma factor antagonist domains have important roles in controlling the activity of sulfate transporters [20, 21]. According to these properties, CRN with sulfate groups is recognized by SLC26A2; thus, it can be well absorbed in the small intestine. Curcumin (Cur), a group of curcuminoids, consists of a seven-carbon linker and three functional groups, including an α,β-unsaturated β-diketone substrate and an aromatic O-methoxy-phenolic group [22]. The keto-form of Cur is able to convert to the enol-form to stabilize itself.

The diketones of keto-Cur are readily deprotonated to enolates because the α,β-unsaturated carbonyl group is a good Michael acceptor and goes through nucleophilic addition [23]. Therefore, Cur is easily soluble in organic solvents but is poorly soluble and less stable in aqueous conditions [24]. However, Cur is known as a potential drug in effective clinical results of antioxidant, anti-inflammatory, and antitumor effects [25, 26]. Cur’s therapeutic effects have been studied in thousands of research papers and more than 120 clinical trials but lack noticeable benefits [27–29]. This is because of their chemical instability, water insolubility, low bioavailability, limited tissue distribution, extensive metabolism, and minimal escape of the gastrointestinal tract [30, 31]. To overcome the limitations of Cur, devising effective approaches to compensate for the poor chemical properties of Cur is an urgent matter.

**Fig. 1:**
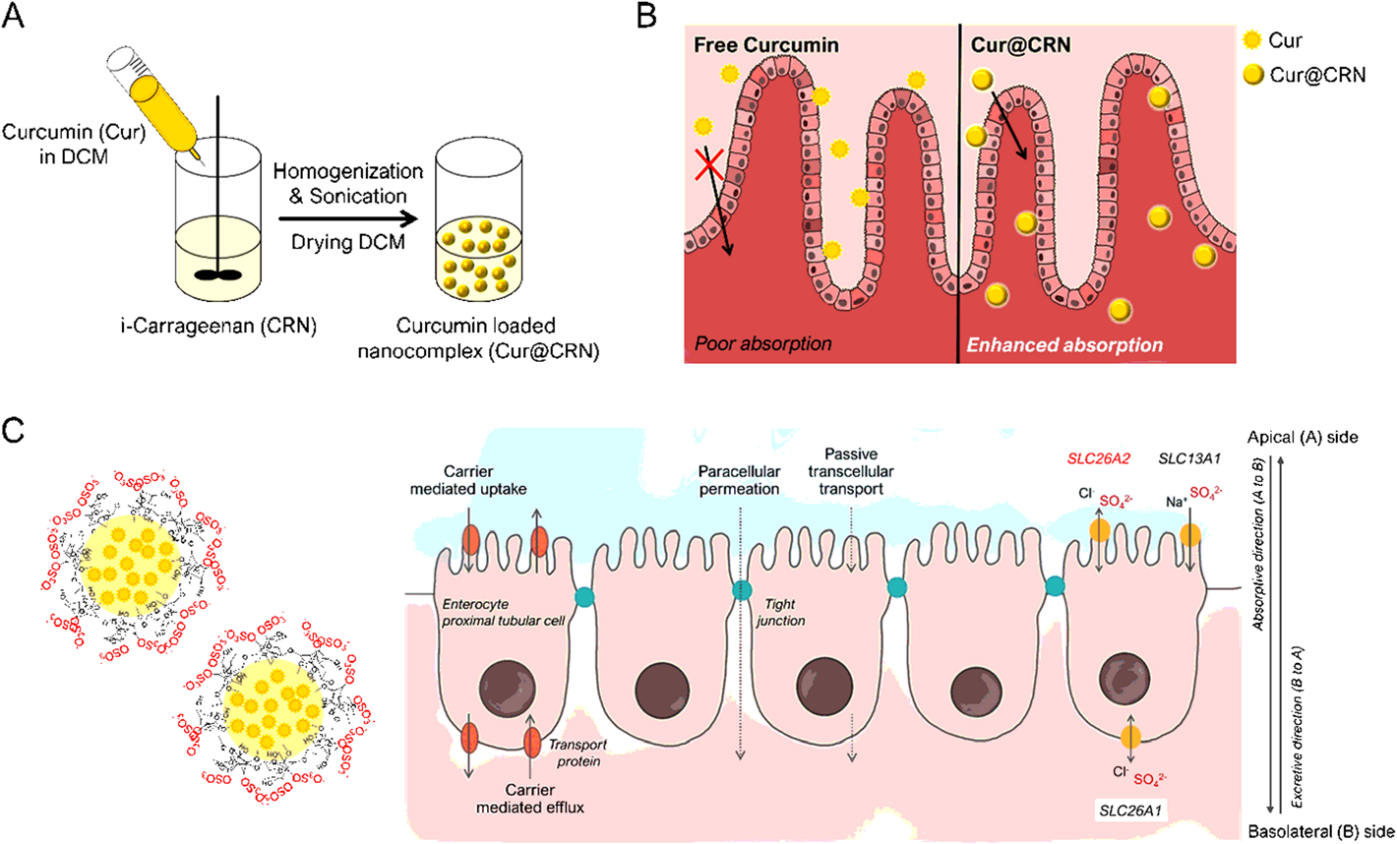
Schematic illustration of the (A) fabrication method and (B) absorption route of orally administered free curcumin or curcumin nanocomposites (Cur@CRN) in the small intestine. (C) Interaction between sulfate groups (SO_4_^2−^) in carrageenan and SLC26A2 that affect active transport

To increase the bioactivity of Cur, many drug delivery systems based on nanotechnology have suitable strategies, such as liposomes, micelles, nanocomposite formations, and nanoemulsion [32]. Emulsion based encapsulation can be easily manufactured, so it is convenient to apply in the academic or industry sectors [33]. Emulsion techniques have excellent performance in protection, controlled release, and site-specific targeted delivery of bioactive components [3]. Cur is solubilized inside the oil droplets, which helps to protect it from chemical degradation by water due to exposure to oxygen and hydroxyl ions [34]. Moreover, water-soluble CRN is wrapped around the Cur droplets to develop suitable formation for oral administration (Fig. 1A and B). We hypothesize that the chemical degradation rate of Cur would be reduced by encapsulation with CRN. Furthermore, CRN promotes the permeabilization of enterocytes and the intestinal barrier, which is achieved by both the high viscosity of CRN and the sulfate recognition of SLC26A2 (Fig. 1C).

Herein, a ι-CRN nanocomposite loaded with Cur (Cur@CRN) was designed via a nanoemulsion method, protecting to chemical and pharmaceutical stability of Cur due to their water-insolubility and hydrophobicity. The stability of Cur in various polymers was confirmed, and the reactive oxygen species (ROS) scavenging efficacy and anti-inflammatory effects of Cur@CRN was shown by blocking the NF-κB/IκBα signaling pathway in vitro. Enhanced intestinal permeability was confirmed through permeability tests in monolayer intestinal cells, and these mechanisms were demonstrated. Overall, Cur@CRN may dismantle the limitations of Cur’s therapeutic effects and may represent a practical platform for use in the clinic and industry.

## Methods

### Materials

Curcumin (Cur) was supplied by JBK-LAB (Seongnam-si, Republic of Korea). ι-carrageenan (CRN), Tween 80 (Tw80), hydroxypropyl cellulose (HPC), 3-(4,5-dimethyl-2-thiazolyl)-2,5-diphenyl-2H-tetrazolium bromide (MTT), and fluorescein-5-isothiocyanate (FITC) were purchased from Sigma-Aldrich (St. Louis, MO, USA). 2,2-Diphenyl-1-picrylhydraqyl (free radical) (DPPH) and Image-iT™ LIVE green reactive oxygen species detection kits were purchased from Invitrogen (Molecular Probes, Inc. Eugene. USA).

### Preparation and characterization of cur@CRN

Cur 10 mg was dissolved in 2 mL of dichloromethane (DCM). Tw80, HPC, and ι-CRN were dissolved in deionized water (D.I water) at concentrations of 0.4% or 0.66% (w/v). The Cur solution was introduced into the Tw80, HPC, and ι-CRN solutions using a syringe, and the mixed solutions were nanoemulsified through homogenization (8000 rpm, 3 min) and ultrasonication (VCS 750, Sonics&Materials, USA) (Cur@Tw80, Cur@HPC, and Cur@ι-CRN). Nanocomposites were stirred at 600 rpm and room temperature (RT) for 24 h to remove DCM. Nanocomposites were then centrifuged at 3000 rpm for 3 min and supernatants were collected to purify free Cur. The loading efficiency (L.E) and loading content (L.C) of nanocomposites were measured using a microplate reader at 425 nm (Bio-Tek, VT, USA). Viscosity was measured using a Brookfield DV2T viscometer (Brookfield engineering laboratories, USA). The hydrodynamic volumes of nanocomposites were measured by a dynamic light scattering (DLS) system (Nanosizer, Malvern Instruments, UK) at 25 °C. The morphology of the nanocomposites was observed under field emission-scanning electron microscopy (FE-SEM, Hitachi, Japan).

Loading efficiency (L.E, %).

= Cur_encapsulated_/Cur_total_ X 100.

Loading content (L.C, %).

= Cur_encapsulated_/Total weight of nanocomposites X 100.

After manufacturing nanocomposites, Cur@Tw80, Cur@HPC, and Cur@CRN were placed in D. I water (pH 6.8) at room temperature to confirm the stability of nanocomposites. Aggregated Cur due to low stability in each sample were removed by centrifugation (3000 rpm, 3 min) each time, and the amount of Cur were confirmed by quantifying Cur for 10 days via a UV/Vis spectrophotometer (UV-2450, 89 Shimadzu, Japan).

### Synthesis of CRN-FITC

Tw80, HPC, and ι-CRN 500 mg and FITC 50 mg were dissolved in D. I water. The mixture was incubated for 24 h (500 rpm). Next, the mixture was precipitated in ethanol and centrifuged at 3000 rpm for 3 min. This process was repeated three more times. After the ethanol was removed in vacuo, the dried powder was dissolved in D. I water and dialyzed for 3 days (MWCO 10 kDa). Finally, Tw80-FITC, HPC-FITC, and ι-CRN-FITC were obtained by lyophilization.

### Cell culture and incubation conditions

Caco-2 (human colon cells, KCLB no.30037.1) and Madin-Darby canine kidney (MDCK, canine kidney cells, KCLB no.10034), and RAW264.7 (mouse macrophage cells, KCLB no.40071) cells were purchased from the Korean Cell Line Bank (KCLB). HaCa-T cells (human keratinocyte) were purchased from the American Type Culture Collection (ATCC, Manassas, Virginia, USA). Fetal bovine serum (FBS), RPMI-1640 medium, antibiotics (penicillin/streptomycin), DMEM (high glucose), and DPBS were obtained from HyClone™ (Logan, Utah 84321, USA). Caco-2 cells were incubated in DMEM containing 1% (v/v) nonessential amino acids (NEAAs), 10% FBS, and 1% antibiotics. MDCK, and HaCa-T cells were cultured in DMEM supplemented with 10% FBS and 1% antibiotics. Cells were cultured at 37 °C in a 5% CO^2^ atmosphere.

### In vitro cell cytotoxicity

Caco-2 cells (1 × 10^4^ cells/well) were seeded in a 96-well plate and allowed to attach for 24 h before sample treatment. Samples (Cur, Cur@Tw80, Cur@HPC, and Cur@CRN, equivalent Cur concentration) were treated with cells for 4 h depending on the concentration. Then, the cells were washed with DPBS, and fresh culture medium was added. After 24 h, the MTT assay was used to confirm cell viability. The absorbance of the MTT dye at 570 nm was detected using a microplate reader (Bio-Tek, VT, USA). The data represent cells viability compared to the negative control (incubated with serum-free medium without samples).

### ROS scavenging activity of cur@CRN

The DPPH assay is generally run to demonstrate antioxidant effects. DPPH solution (3.9 mL, 0.025 mg/mL) in methanol was mixed with the sample solution (0.1 mL). The reaction progress absorbance of the mixture was monitored at 515 nm for 30 min or until the absorbance was stable. Upon reduction, the color of the solution fades from purple to yellow. The percentage of the remaining DPPH was calculated as %DPPH_remained_, as shown equation.

%DPPH_remained_ = [DPPH]_remained_/[DPPH]_initial_ X 100%DPPH_remained_ is proportional to the antioxidant concentrations, and the concentration that causes a decrease in the initial DPPH concentration by 50% is defined as the EC50. The time needed to reach the steady state with EC50 concentration was calculated from the kinetic curve and was defined as T_EC50_.

To validate intracellular ROS scavenging effects, HaCa-T cells were seeded in black 96-well plates at 1 × 10^4^ cells/well for 24 h and were incubated with Cur, Cur@Tw80, Cur@HPC, and Cur@CRN (5 μg/mL, Cur equivalent) for 4 h. Tert-butyl hydrogen peroxide (tBHP) as the ROS inducer was preincubated for 30 min. Cells were washed with DPBS and stained with 2′,7′–dichlorofluorescein diacetate (DCF-DA, 25 μM) in DPBS for 30 min in the dark at 37 °C. Fluorescence was estimated using a microplate reader (Ex/Em = 485/535 nm).

### Western blot

Western blot analysis was performed to measure the inflammatory factors induced by lipopolysaccharide (LPS, 10 μg/mL), LPS + Cur, LPS + Cur@Tw80, LPS + Cur@HPC, and LPS + Cur@CRN (5 μg/mL, Cur equivalent). HaCa-T and RAW264.7 cells were incubated with samples for 4 h. Total protein was collected and lysed using RIPA buffer (Pierce, Rockford, IL, USA) containing a protease inhibitor cocktail (Roche, Germany) and phosphatase inhibitor cocktail 3 (Sigma-Aldrich, St. Louis, MO, USA). Total proteins (30 μg) were separated on 10% (w/v) acrylamide SDS-PAGE gels by electrophoresis and were transferred onto polyvinylidene difluoride (PVDF) membranes using a semidry system (Trans Blot® SD Semi-Dry Transfer Cell, Bio Rad, California, USA). The membranes were blocked in 5% skim milk or 5% BSA and then incubated with each antibody. The primary antibodies used in our study included: anti-NF-κB (1:200; ab16502, Abcam), anti-IκBα (1:1000; ab32518, Abcam), and anti-p-IκBα (1:10000; ab133462, Abcam). A monoclonal antibody against anti-β-actin (1:5000; BA3R, Thermo Fisher Scientific) served as a loading control. A horseradish peroxidase (HRP)-conjugated secondary antibody (Santa Cruz Biotechnology, CA, USA) was used to amplify the signal. Protein signals were detected using a chemiluminescence system (Bio Rad, California, USA).

### Permeability test of cur@CRN

Permeability experiments were carried out using transwell inserts (polycarbonate membrane, 0.4 μm pore size, 24 mm diameter, Corning Inc., Corning, NY). Inserts were placed in 6-well plates. MDCK and Caco-2 cells were seeded at 1 × 10^5^ cells/well on the membrane inserts with 1.5 mL of medium (the apical side, A), and then the basolateral side (B) was filled with 2.5 mL of medium. Cells were grown and differentiated into confluent monolayers for 4 or 21 days. Transepithelial electrical resistance (TEER) in the transwell was measured using a Millicell-ERS voltmeter (Millipore Co., Billerica, MA). A monolayer was used in the experiment when it had a TEER value higher than 250 Ω cm^2^. To progress the bidirectional permeability assay, all samples (Cur, Cur@Tw80, Cur@HPC, and Cur@CRN) in Hank’s balanced salt solution (HBSS) were added to the apical side or the basolateral side for 2 h (150 μM, Cur equivalent). Cur levels in the apical and basolateral sides were measured using LC-MS/MS. The LC-MS/MS system consisted of an Agilent 1260 HPLC system (Agilent Technologies, Wilmington, DE, USA) coupled to a triple quadrupole API 5500 mass spectrometer (AB SCIEX, Foster City, CA, USA) equipped with an electrospray ionization interface operated in negative ion mode. Chromatographic separation was performed using a Poroshell 120 EC-C18 column (4.6 × 50 mm, 2.7 μm; Agilent Technologies, Santa Clara, CA) maintained at 40 °C. The mobile phase consisted of 0.1% formic acid in D. I water (A) and 100% acetonitrile (B) (20:80, v/v) at a flow rate of 0.45 mL/min. The retention time for Cur and chlorpropamide were both 1.4 min, and the observed transition were m/z 367.15 > 149.10, and 274.90 > 189.60, respectively. The calibration curves were linear (r^2^ ≥ 0.992) over the concentration range of 0.5–200 ng/mL for Cur. The within- and between-batch accuracies and levels of precision were within the acceptable limits of ±15%. The permeability indicates the permeability coefficient (Papp), which utilizes the efflux ratio [Papp(B-A)/Papp(A-B)].

$$ Papp=\frac{V_r\times {dC}_r}{dt\times A\times {C}_{D0}} $$

V_r_: the volume of the receiver compartment.

dC_r_/dt: the change in concentration of the compound in the receiver compartment over time.

A: the area of the permeability barrier.

C_D0_: the initial compound concentration in the donor compartment.

### In vitro absorption mechanism study via SLC26A2

AGS (human gastric), HuTu-80 (human duodenum), Caco-2 (human colon), MDCK (canine kidney), HaCa-T (human keratinocyte), and L929 (mouse fibroblast) cells were seeded at 1 × 10^5^ cells/tube in round-bottom tubes. The cells were fixed using 4% paraformaldehyde in DPBS pH 7.4 for 10 min at room temperature. After washing, the cells were incubated with 1% BSA, 22.52 mg/mL glycine in PBST (DPBS and 0.1% (v/v) Tween 20) for 30 min to block nonspecific binding of antibodies. The cells were stained with anti-SLC26A2 (1:200; 27,759–1-AP, Proteintech group) and anti-rabbit Alexa Fluor 594 (1:200; A120-101D4, Bethyl laboratories) at 4 °C. Following washing, stained cells were analyzed using a Becton-Dickinson FACS Canto II instrument (San Jose, CA, USA). Each experiment was analyzed statistically using FACS Diva software (BD).

Caco-2, L929, and MDCK cells were seeded at 2 × 10^5^ cells/well in 6-well plates for 24 h. Samples (Tw80-FITC, HPC-FITC, and CRN-FITC) were treated with cells for 2 h (40 μg/mL, FITC equivalent). After the samples were washed out, the cells were fixed with 4% paraformaldehyde and stained with anti-SLC26A2 (1st Ab, 1:200; 27759–1-AP) and anti-rabbit (2nd Ab, 1:200; A120-101D4) antibodies. Cells were stained with 4′,6-diamidino-2-phenylindole (DAPI) to visualize nuclei. FITC was captured using a confocal laser scanning microscope (CLSM, Zeiss LSM 710 Meta, Oberkochen, Germany). Analysis of the images was performed via LSM Image Browser software (Zeiss, Germany, USA).

Caco-2 cells (1 × 10^6^ cells/well in 6-well plates) were incubated with FITC-labelled CRN (concentration of FITC, 2 μg/mL) alone or combination with various polysaccharides (i-CRN, Chondroitin sulfate, HPC, and Hyaluronic acid) for 1 h at 37 °C to verify the competitive inhibition test. The cells were rinsed, harvested, and re-suspended in DPBS. All samples were quantitatively analyzed using flow cytometer with FITC channel. Also, cellular internalized Cur@CRN was dissolved in lysis solvent (ethanol: DPBS (0.1% triton X)) and detected with UV spectrometer at 420 nm.

## Statistical analysis

The results are presented as the means ± standard deviation for results obtained from three independent trials, unless indicated otherwise. The results from the ex vivo and in vivo studies are expressed as the mean ± standard error of the mean (SEM). Statistical significance was determined using one-way analysis with *p*-values < 0.05 established as the minimal level of significance. (∗*p* < 0.05, ∗∗*p* < 0.01, and ∗∗∗*p* < 0.001).

## Results

### Synthesis and characterization of OPuC

**Fig. 2:**
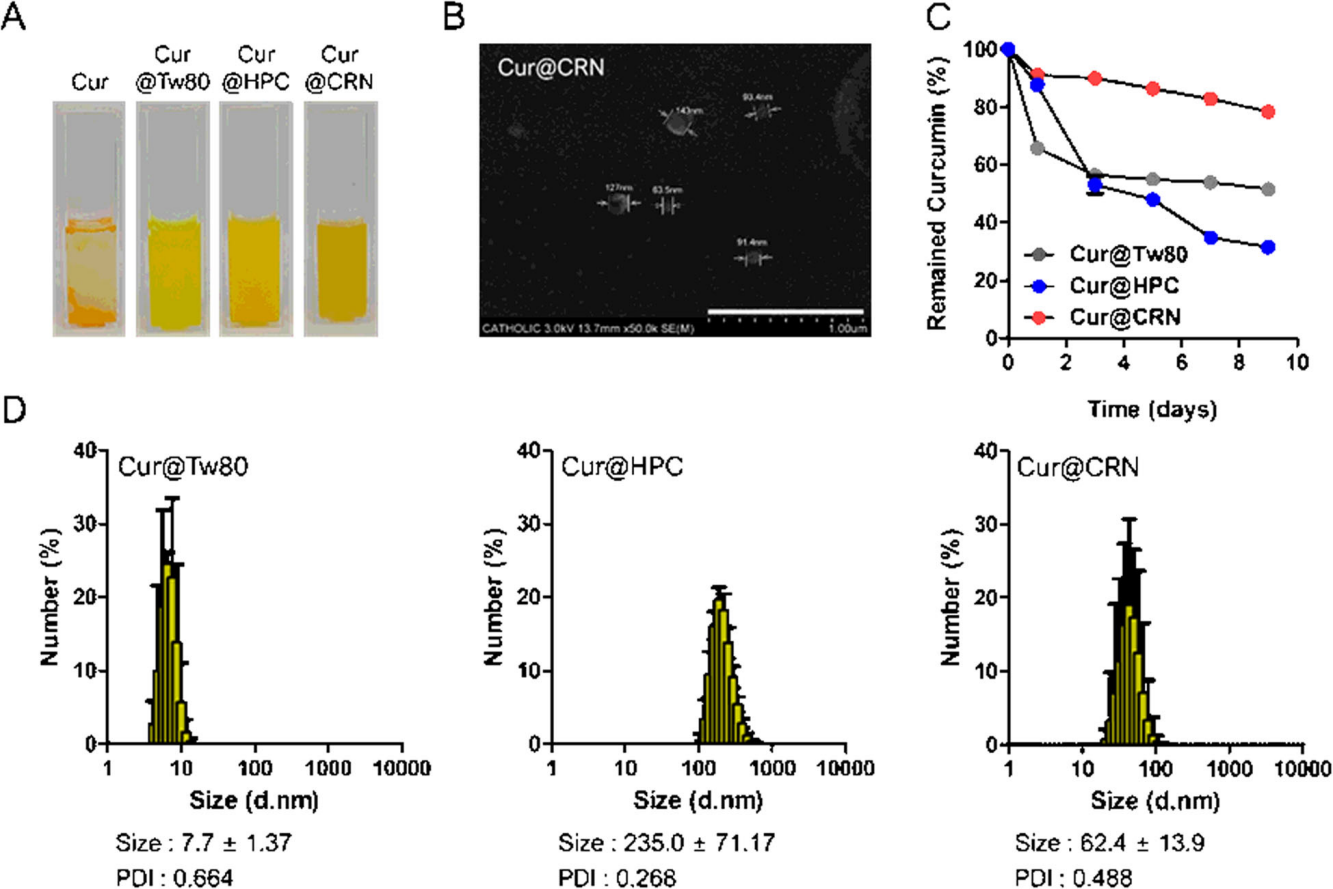
Characterization of Cur@CRN. (A) Optical images after Cur emulsification. (B) SEM images of Cur@CRN (3.0 kV, scale bar = 1.00 μm) (C) Stability of Cur@Tw80, Cur@HPC, and Cur@CRN for 9 days confirmed by UV-vis spectrometry (at 425 nm). (D) Hydrodynamic size of Cur@Tw80, Cur@HPC, and Cur@CRN after emulsification

Before confirming the characterization of nanocomposites, the viscosity of polymers to be used in composites was measured. The viscosity of the free polymer (Tw80, HPC, and ι-CRN) was measured at 0.40% or 0.66% (w/v) (Supplement Table S1). Tw80 and HPC showed ambiguous differences depending on the concentration, but it was confirmed that the viscosity of ι-CRN significantly increased at 0.66%. In general, the more increased the polymer concentration, the more the loading efficiency (L.E.) improved apart from the loading content (L.C.). The encapsulation efficiency of Cur was enhanced by optimizing various volume conditions of water dissolved in the polymer while keeping the weight ratio of Cur:CRN constant at 1:10. As a result, nanocomposites made of CRN indicated higher L. E and L. C due to the higher viscosity of CRN compared with Tw80 and HPC (Table 1). Additionally, the viscosity of the nanocomposites was conspicuously increased compared with other nanoparticles (Supplementary Table S2). Developed nanocomposites were well-dispersed in D. I water due to the improved solubility using hydrophilic polymers, even though Cur is water-insoluble (Fig. 2A).

**Table 1:** Loading efficiency (L.E) and Loading content (L.C) of curcumin-loaded nanocomplexes (Cur@X; X indicates various polymers)

Cur@X(Cur 10 mg)	0.66% (w/v, 15 mL)	
	Loading efficiency (%)	Loading content (%)
**Tw80**	56.81 ± 4.06	6.53 ± 0.15
**HPC**	62.21 ± 4.01	8.51 ± 0.15
**CRN**	93.02 ± 5.65	8.89 ± 0.19

The formation of nanocomposites was confirmed via hydrodynamic size and SEM images. As shown Fig. 2B, the size of Cur@CRN was approximately 103.66 nm. In addition, the sizes of Cur@Tw80, Cur@HPC, and Cur@CRN were determined to be 7.7 ± 1.37, 235.0 ± 71.17, and 62.4 ± 13.90, respectively (Fig. 2D). To confirm the stability of the nanocomposites, the residual amount of Cur was tracked for 9 days. Cur@CRN was most stable for 10 days due to its comparatively high viscosity, but the Cur remaining in Cur@Tw80 and Cur@HPC rapidly decreased (Fig. 2C).

### In vitro cytotoxicity and antioxidant efficacy

**Fig. 3:**
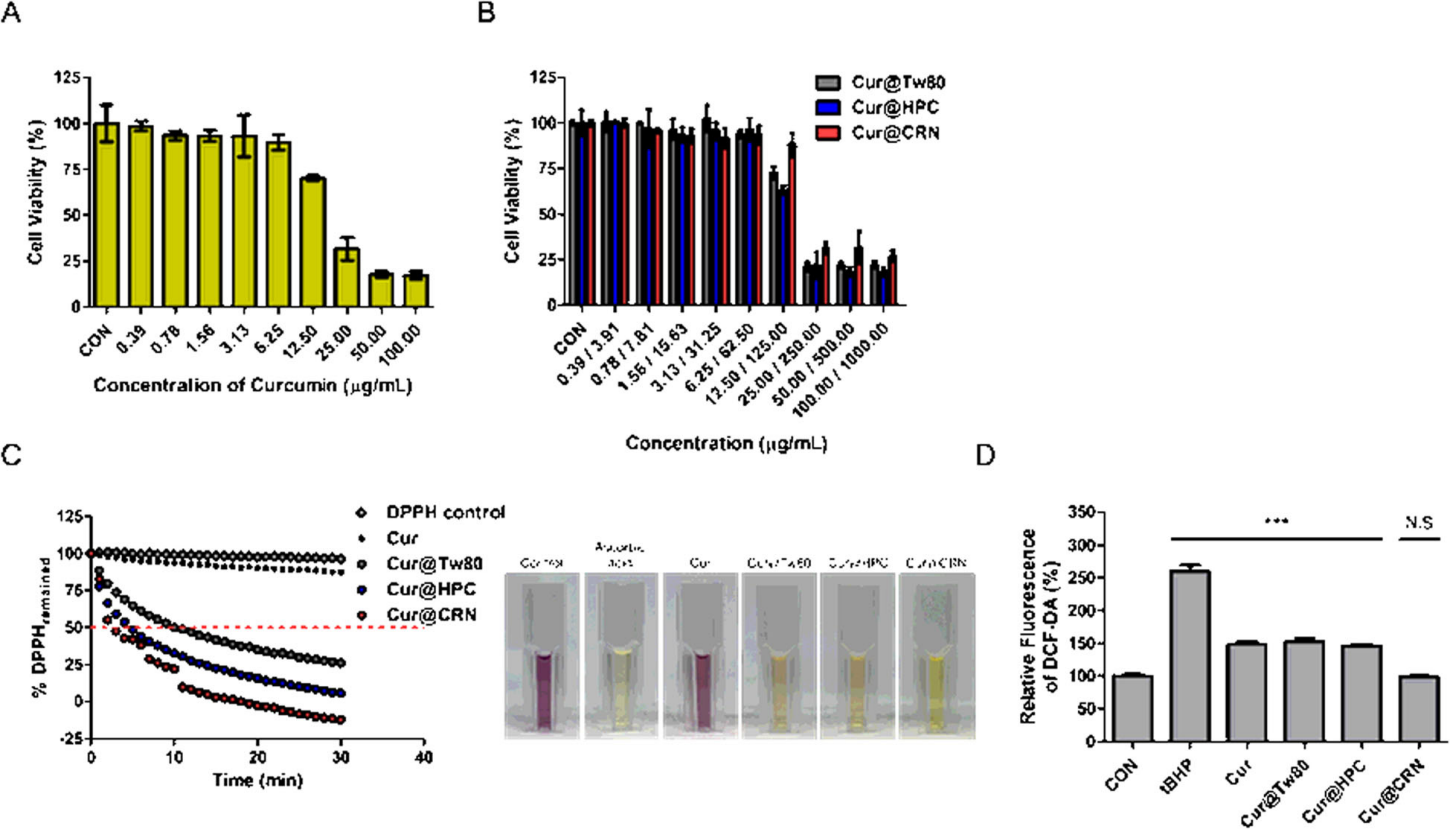
In vitro cytotoxicity against Caco-2 cells after (A) Cur, (B) Cur@Tw80, Cur@HPC, and Cur@CRN treatment for 4 h. (C) DPPH bleaching kinetics in the presence of Cur, Cur@Tw80, Cur@HPC, and Cur@CRN (2 μg/mL, Cur equivalent) at 515 nm for 30 min. Images of the colorimetric change from purple to yellow (2 μg/mL ascorbic acid, positive control). (D) Intracellular fluorescence of DCF-DA after incubation with tBHP (positive control), Cur, Cur@Tw80, Cur@HPC, and Cur@CRN (5 μg/mL, Cur equivalent) (*n* = 3)

The cytotoxicity of free Cur, Cur@Tw80, Cur@HPC and Cur@CRN in Caco-2 cells were demonstrated with an MTT assay. Caco-2 cells incubated with Cur rarely exhibited evidence of toxicity until a concentration of 6.25 μg/mL, and the viability gradually decreased according to increasing concentrations (Fig. 3A). Based on this result, treatment with Cur@Tw80, Cur@HPC, and Cur@CRN hardly induced any cytotoxicity until the concentration of 6.25 μg/mL (Fig. 3B). One notable observation is that the viability of Cur@CRN was approximately 20.27% higher than those of the other groups at 12.50/125.00 (Cur/polymer) μg/mL. In addition, we confirmed the cytotoxicities of various types of CRN (*i.e.*, ι-CRN high molecule, ι-CRN low molecule, κ-CRN, and λ-CRN), Tw80, and HPC. All CRNs were found to be safe for Caco-2 cells (Supplement Fig. S1 and S2).

Nanocomposites made of various types of CRN (*i.e.*, Cur@ι-CRN high molecule, Cur@ι-CRN low molecule, Cur@κ-CRN, and Cur@λ-CRN) rarely caused cell death until concentrations of 12.50/125.00 (Cur/CRN) μg/mL. At concentrations of 12.50/125.00 (Cur/CRN) μg/mL or more, the cell viability was significantly reduced, like the results of the Cur cytotoxicity test.

To confirm antioxidant efficacy, we implemented DPPH and DCF-DA assays. DPPH is a long-lived nitrogen radical kinetic behavior of the compound, so it was suitable for quickly confirming that antioxidants react with peroxyl radicals, as evident from the T_EC50_ values (red line) ranging from 0 to 30 min or colorimetric change from purple to yellow (Fig. 3C). Ascorbic acid (positive control) turned yellow as soon as it was exposed to DPPH. After incubating the sample and DPPH for 30 min, Cur@Tw80, Cur@HPC, and Cur@CRN changed yellow, but no color change was observed with Cur. In Fig. 3C, Cur@CRN represented the steepest decreasing slope, reaching its T_EC50_ faster than the other formulations. It was possible to exhibit a sufficient role of ROS scavenging in aqueous conditions because Cur’s poor solubility was addressed through formulations with polymers. To demonstrate that this antioxidant effect is not due to the polymer (Tw80, HPC, and CRN), the same experiment was conducted using free polymers (Supplement Fig. S3).

Similarly, a DCF-DA assay was performed to demonstrate the intracellular antioxidant effects. tBHP (positive control) was utilized to arbitrarily increase the intracellular ROS levels. Subsequently, HaCa-T cells were incubated with Cur, Cur@Tw80, Cur@HPC and Cur@CRN (5 μg/mL, Cur equivalent) for 4 h. The reduced fluorescence intensity of DCF-DA indicates decreased ROS levels by Cur. All samples exhibited low fluorescence, that they successfully scavenged ROS as antioxidant agents (Fig. 3D). Despite Cur being coated with a high-molecular-weight polymer, the ROS-scavenging ability was maintained in the intracellular system. In Cur@CRN, the fluorescence was reduced by approximately 62.24% compared with tBHP, whereas the fluorescence of the other groups was reduced approximately 42.88%.

Taken together, Cur@CRN indicated the greatest reduction in ROS because CRN not only enables the high stability and dispersibility of Cur but also because CRN itself has free radical scavenging properties [35]. Therefore, the combination of two antioxidants, Cur and CRN, can be expected to have synergistic effects, which would be effectively interfere with the inflammatory pathway.

### In vitro anti-inflammatory effects

**Fig. 4:**
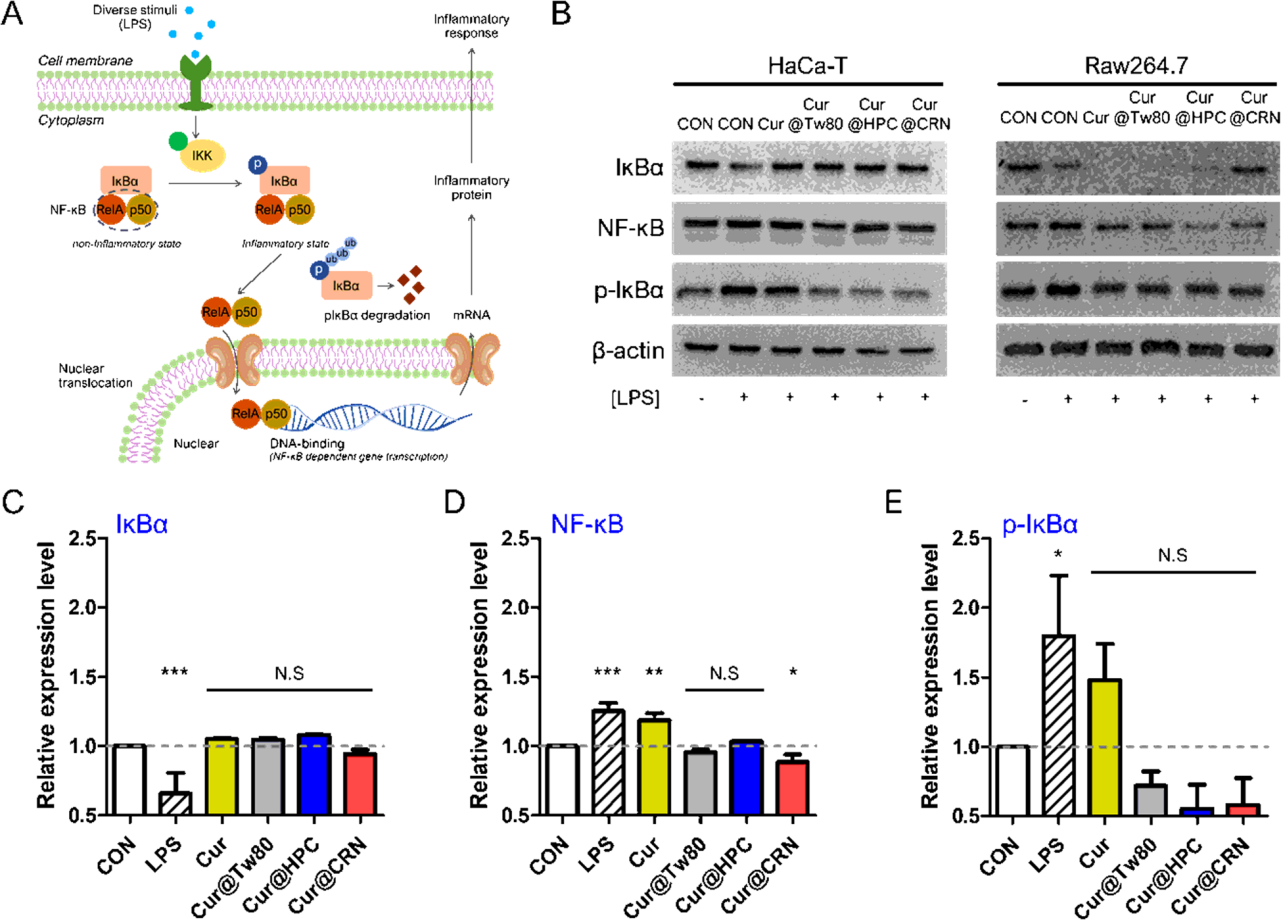
In vitro anti-inflammatory effects of Cur@CRN. (A) Illustration of the NF-κB/IκBα signaling pathway. (B) Representative western blotting bands for IκBα, NF-κB, and phosphorylated(p)-IκBα in HaCa-T and RAW264.7 cells. (C-E) Relative protein levels of inflammation-related factors in HaCa-T cells (based on Fig. 4B)

Inflammation is the process of innate immunity in response to oxidative stress and is associated with activation of the NF-κB signaling pathway [36]. Antioxidants prevent unwanted inflammatory responses because they protect tissues from damage. Anti- inflammatory effects were confirmed by the NF-κB/IκBα signaling pathway [37]. NF-κB is a central mediator of proinflammatory gene induction and functions in both innate and adaptive immune cells. IκBα binds to NF-κB subunits where it is thought to interact with the NF-κB DNA binding and nuclear localization region, preventing NF-κB translocation to the nucleus [38]. Upon activation, the IκB kinases phosphorylate the inhibitory IκB proteins bound to NF-κB (Fig. 4A). Based on this mechanism, representative factors (total NF-κB, pIκBα, and total IκBα) were chosen to demonstrate the anti-inflammatory effects of Cur [39].

To confirm the anti-inflammatory effects of Cur, HaCa-T cells were incubated with LPS to artificially induce an inflammatory environment. The effects of LPS on NF-κB and inflammatory mediators have been well characterized as a common inflammatory stimulus [40].

Following treatment with LPS, IκBα triggers ubiquitin (ub)-dependent degradation and phosphorylation by IκB kinases. These phenomena were confirmed by western blotting (Fig. 4B), indicating decreased expression levels of IκBα and increased expression levels of both NF-κB and pIκBα (Fig. 4C-E). However, incubation with the formulated Cur maintained the NF-κB/IκBα complex, which was demonstrated by the relative expression levels of IκBα and NF-κB, like the negative control group. Furthermore, Cur@CRN significantly reduced the expression of pIκBα (increased due to LPS), although Cur presented a negligible difference from LPS (Fig. 4E). Taken together, the results indicate that the improved stability and solubility of Cur could enhance its role as an antioxidant, thereby exerting an effective anti-inflammatory effect.

### In vitro permeability test and targeting ability

**Fig. 5:**
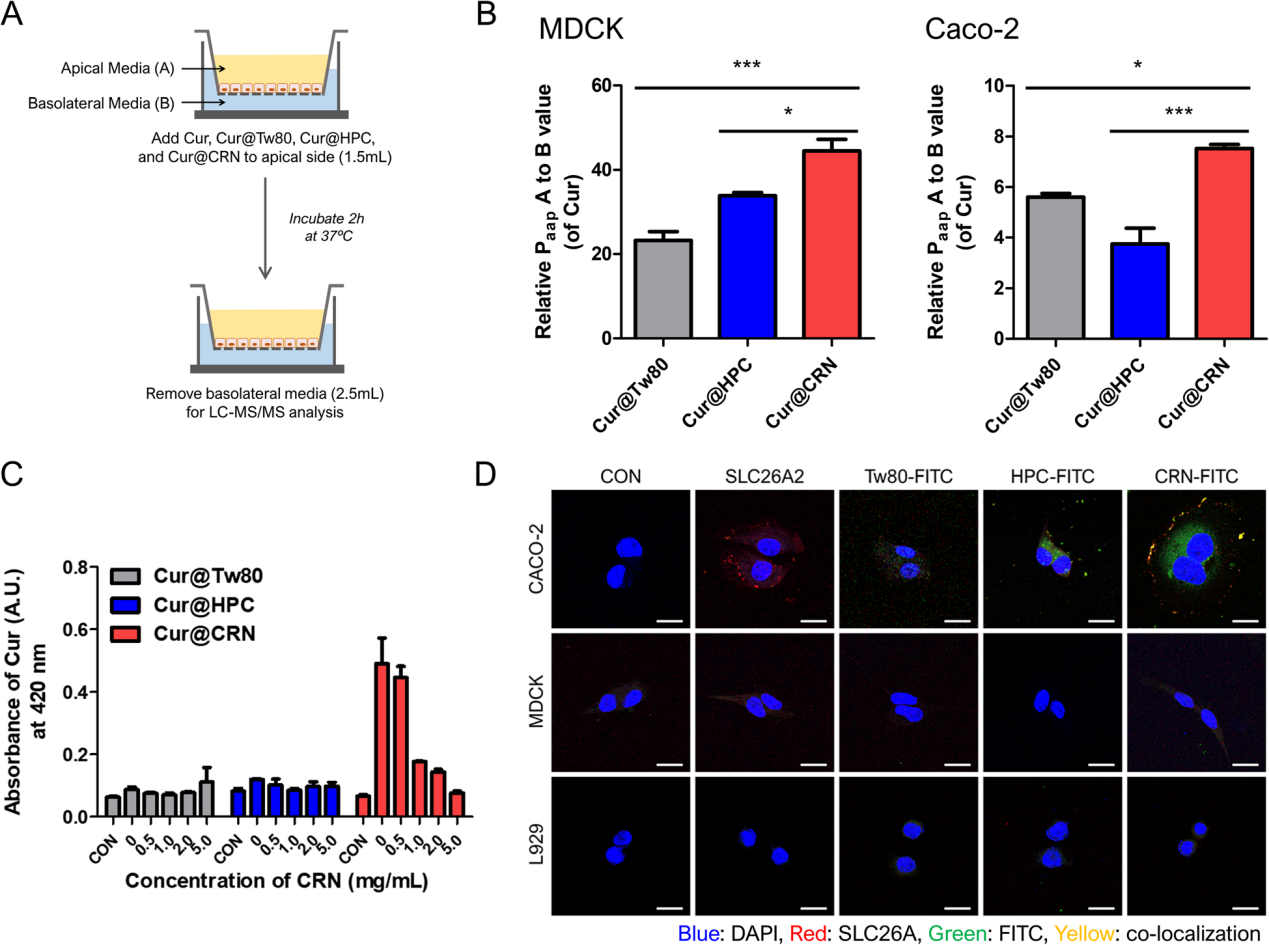
In vitro permeability and absorption mechanism of Cur@CRN. (A) Schematic process of the permeability test via the transwell system. (B) Permeabilities of various compounds across the MDCK and Caco-2 cell monolayers (Paap values are presented in Supplementary Table S2, n = 3). (C) Competitive inhibition test by co-incubating free CRN with various nanocomposites (n = 3). (D) Confocal images of colocalization between SLC26A2 and Tw80-FITC, HPC-FITC, and CRN-FITC in various cells (5 μg/mL, Cur equivalent) (starting from the top panel, Caco-2; positive cells, L929 and MDCK; negative cells)

Caco-2 (human colon cells) and MDCK (canine kidney cells) cells are used to measure permeability and absorption in drug discovery studies. Caco-2 and MDCK cells show similar magnitudes of passive transcellular transport [41, 42]. However, Caco-2 cells are densely arranged and have higher expression levels of transporters, including active facilitated transport, than MDCK cells, including SLC26A2 [43, 44]. Cell monolayers mimic the intestinal epithelium, and transport assessment used as an indicator of drug intestinal absorption can be performed in two directions: apical to basolateral or basolateral to apical (Fig. 5A). The permeabilities of all groups (Cur@Tw80, Cur@HPC, and Cur@CRN) were presented by calculating the Paap values according to the movement direction of the sample (Fig. 5B). The more permeable the cell layer, the lower the efflux ratio due to an increase in movement from the apical to basal layer (Supplement Table S3). In Caco-2 and MDCK cells, the nanocomposites made of Tw80, HPC, and CRN enhanced cell permeability compared with free Cur. Among them, Cur@CRN exhibited the highest relative Paap A to B ratio in both MDCK cells (44-fold) and Caco-2 cells (8-fold), which means successful intestinal cell layer penetration. Notably, Caco-2 cells exhibited significantly higher Papp values than MDCK cells (Supplement Table S3). These results indicate that the permeability of MDCK cells is the result of only passive transport, but that of Caco-2 cells is the result both passive and active transport. Therefore, the Cur@CRN transport process can protect against carrier-mediated active transport.

We demonstrated why Cur@CRN penetrated the cell layer more than other polymer composites instead of CRN. This is because we hypothesized that CRN has many sulfates functional groups. Solute carrier family 26 member 2 (SLC26A2), which is the sulfate (SO_4_^2−^) transporter, accepts chloride, hydroxyl ions (OH^-^), and oxalate as substrates [45]. As a result of comparing the expression levels of SLC26A2 in various cells, it was mainly expressed in gastrointestinal cells, of which Caco-2 cells were the most prominent (Supplement Fig. S4). SLC26A2, located on the surface of Caco-2 cells (positive cells), was effectively colocalized with ι-CRN-FITC in confocal microscopic images (Fig. 5D). Furthermore, it was difficult to determine the fluorescence of the stained SLC26A2 and FITC in MDCK and L929 cells (negative cells). These results not only support the in vitro permeability test but also indicate the increased adsorption of Cur to the small intestinal wall via CRN.

To support this mechanism, we proved additional competitive inhibition test by co-incubating with excess sulfate group (Fig. 5C). The Caco-2 cells treated with only CRN (0.5, 1.0, 2.0, and 5.0 mg/mL) for 1 h and subsequently incubated with Cur@Tw80, Cur@HPC, and Cur@CRN (Conc. 5 μg/mL of Cur). Cur@CRN group which was non-incubated with CRN presented high level curcumin, but Cur@CRN were not internalized as the concentration of CRN increases because free CRN interacted with SCL26A. However, these phenomena were not presented in the other groups (Cur@HPC and Cur@Tw80).

In a similar way, the Caco-2 cells was pre-incubated with the various types of polymers (sulfate positive; CRN and Chondroitin sulfate, sulfate negative; Hydroxypropyl cellulose and Hyaluronic acid), and then incubated with FITC-labelled CRN (Supplement Fig. S5). In CRN and Chondroitin sulfate groups (positive control), the higher co-incubated polymer concentration, the lower FITC intensity because of the competitive inhibition via SLC26A and sulfate. But, in Hydroxypropyl cellulose and Hyaluronic acid group, the intensity of FITC was similar any concentration of co-incubated polymers. Therefore, the enhanced permeability of Cur@CRN was demonstrated by confirming the interaction of SLC26A and sulfate group.

## Discussion

To improve the bioavailability of Cur, CRN is most suitable candidate than other polymers. The viscosity of CRN is a major factor influencing the improvement of stability, loading efficiency, and loading contents. Cur@CRN accomplished the most dispersibility, exhibiting the greatest antioxidant effects and the long-term stability. Antioxidant capacity was confirmed by the DPPH method for the nanocomposites itself, and the intracellular efficacy confirmed by DCF-DA assay. The high antioxidant effect was directly related to the anti-inflammatory effects. In addition, Cur entered the cells and acted as a blockade of NFκB/IκBα signaling pathway. Therefore, CRN is appropriate to encapsulate unstable Cur, improving its drug stability and bioactivity in oral administration.

In Caco-2 cells, the polymers (all types of CRN, Tw80, and HPC) used in the nanocomposites are biocompatible materials, indicating cytotoxicity through MTT assay. Toxicity of nanocomposites was shown at the Cur standard concentration of 6.25 μg/mL or higher, which is like the results of free Cur itself. Cur-nanocomposites enable to penetrate across cell monolayer via active or passive permeability pathway. Cur@CRN injected in apical side well was more penetrated to basal side well. This is because, CRN has sulfate groups, which enable to interact SLC26A2 receptors. SLC26A2 include in the active intestinal transport pathway, which overexpressed intestine.

## Conclusion

Nanocomposites loaded with curcumin and carrageenan (Cur@CRN) were developed to enhance the bioavailability of Cur, which is insoluble and has low stability under water conditions. Cur was well loaded in CRN, and its stability was maintained for a long time compared to other polymers due to its comparatively high viscosity. Cur has anti-inflammatory effects via its antioxidant effects, but it cannot show sufficient effects due to its physical limitations. However, Cur formulated with CRN showed dramatic antioxidant effects, which were confirmed in vitro by its enhanced intracellular ROS-scavenging effects. Additionally, since CRN has antioxidant and anti-inflammatory properties, Cur@CRN exhibited more anti-inflammatory synergistic effects, as confirmed by the NF-κB/IκBα pathway. In addition, CRN is sulfate-containing polymer that proactively interacts with the SLC26A2 transporter, which a part of the solute carrier expressed in intestinal cells. Therefore, it was revealed that Cur loaded in CRN can effectively penetrate the intestinal barrier via the active transport process of SLC26A2. As a result, the high viscosity of CRN and its interaction with SLC26A2 enable to maximize the intestinal permeability. Further, Cur@CRN would be confirmed in vivo pharmacokinetics parameter to demonstrate the enhanced bioavailability of orally taken Cur. Assessing from this research, the platform with carrageenan could promote the oral bioavailability of any drug which has low solubility and poor stability in water (but also Cur). This CRN platform serves as a novel and easy approach to promote oral medications.

## Supplementary Information

**Additional file 1.**

## Data Availability

Data sharing is not applicable to this article.

## Abbreviations

CRN: Carrageenan
Cur: Curcumin
SLC26: Solute carrier 26
Tw80: Tween 80
HPC: Hydroxypropyl cellulose
DPPH: 2,2-Diphenyl-1-picrylhydraqyl (free radical)
L.E: Loading efficiency
L.C: Loading content
