# Supplementary material for: ι-Carrageenan nanocomposites for enhanced stability and oral bioavailability of curcumin
Source: Biomater Res. 2021 Oct 9;25:32. doi: 10.1186/s40824-021-00236-4 (PMC8502325; doi:10.1186/s40824-021-00236-4)
Supplement: Supplementary file 1 — Additional file 1. [file 40824_2021_236_MOESM1_ESM.docx]

*Supplementary data*

**ι-Carrageenan nanocomposites
for enhanced stability and oral-bioavailability of curcumin**

*Joo Young Lee ^a, 1^, Sanghee Lee ^a, 1^, Jang Ho Choi and Kun Na ^a,^ **

JY. Lee, S. Lee, JH. Choi, Prof. K. Na

^a^Department of Biotechnology, Department of Biomedical-Chemical Engineering,

The Catholic University of Korea, 43 Jibong-ro, Wonmi-gu, Bucheon-si, Gyeonggi-do, 14662, Republic of Korea

* **Corresponding author**: Kun Na, Ph.D.

Department of Biotechnology, Department of Biomedical-Chemical Engineering,
The Catholic University of Korea, 43 Jibong-ro, Wonmi-gu, Bucheon-si, Gyeonggi-do, 14662, Republic of Korea

^1^These authors contributed equally to this work.

**Tel.:** +82-2-2164-4832

**Fax.:** +82-2-2164-4865

**E-mail:** [kna6997@catholic.ac.kr](mailto:kna6997@catholic.ac.kr)

(mPa·s)

|  | **RPM**  **Conc. (%)** | **50** | **100** | **200** |
| --- | --- | --- | --- | --- |
| **Tw80** | 0.40% | 9.00 | 3.50 | 2.50 |
|  | 0.66% | 7.00 | 3.00 | 2.50 |
| **HPC** | 0.40% | 8.00 | 5.50 | 2.50 |
|  | 0.66% | 11.00 | 6.00 | 4.00 |
| **ι-CRN** | 0.40% | 72.00 | 45.50 | 33.50 |
|  | 0.66% | 297.00 | 188.50 | 142.00 |

**Table S1.** Viscosity of free polymer at room temperature using rotating spindle number 21 at 25 ºC.

(mPa·s)

|  | **RPM**  **Conc. (%)** | **10** | **20** | **50** | **80** | **100** | **120** | **200** |
| --- | --- | --- | --- | --- | --- | --- | --- | --- |
| **Tw80** | **0.40%** | 35.00 | 17.50 | 9.00 | 3.14 | 3.50 | 3.33 | 2.50 |
|  | **0.66%** | 20.00 | 5.00 | 7.00 | 7.50 | 3.00 | 3.75 | 2.50 |
| **HPC** | **0.40%** | 30.00 | 7.50 | 8.00 | 6.10 | 5.50 | 4.17 | 2.50 |
|  | **0.66%** | 10.00 | 12.50 | 11.00 | 6.88 | 6.00 | 5.00 | 4.00 |
| **ι-CRN** | **0.40%** | 145.00 | 110.00 | 72.00 | 56.30 | 45.50 | 42.50 | 33.50 |
|  | **0.66%** | 880.00 | 557.80 | 297.00 | 222.00 | 188.50 | 167.50 | 142.00 |

**Table S2.** Viscosity of a various type of nanocomposites at room temperature using rotating spindle number 21 at 25 ºC.


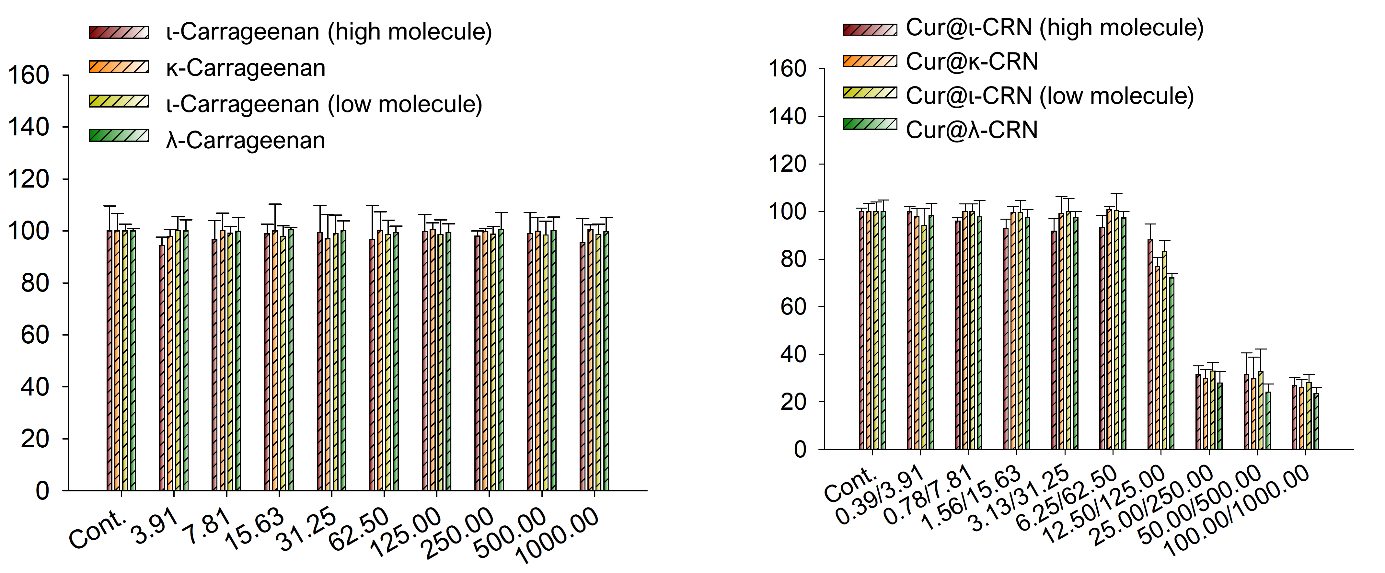


**Fig. S1.** *In vitro* cytotoxicity against Caco-2 cells after incubation with a various type of carrageenan and their nanocomplex with Cur. Concentration of Cur@CRN presented for curcumin/carrageenan with various contents.


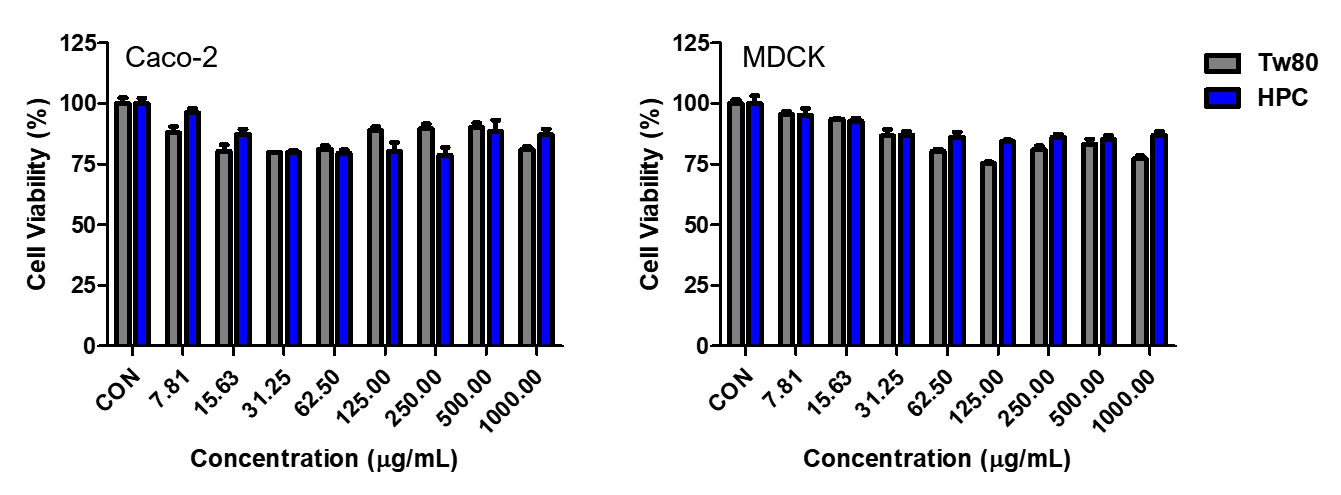


**Fig. S2.** *In vitro* cytotoxicity against Caco-2 and MDCK cells after incubation with a various type of Tween80 and Hydroxypropyl cellulose.


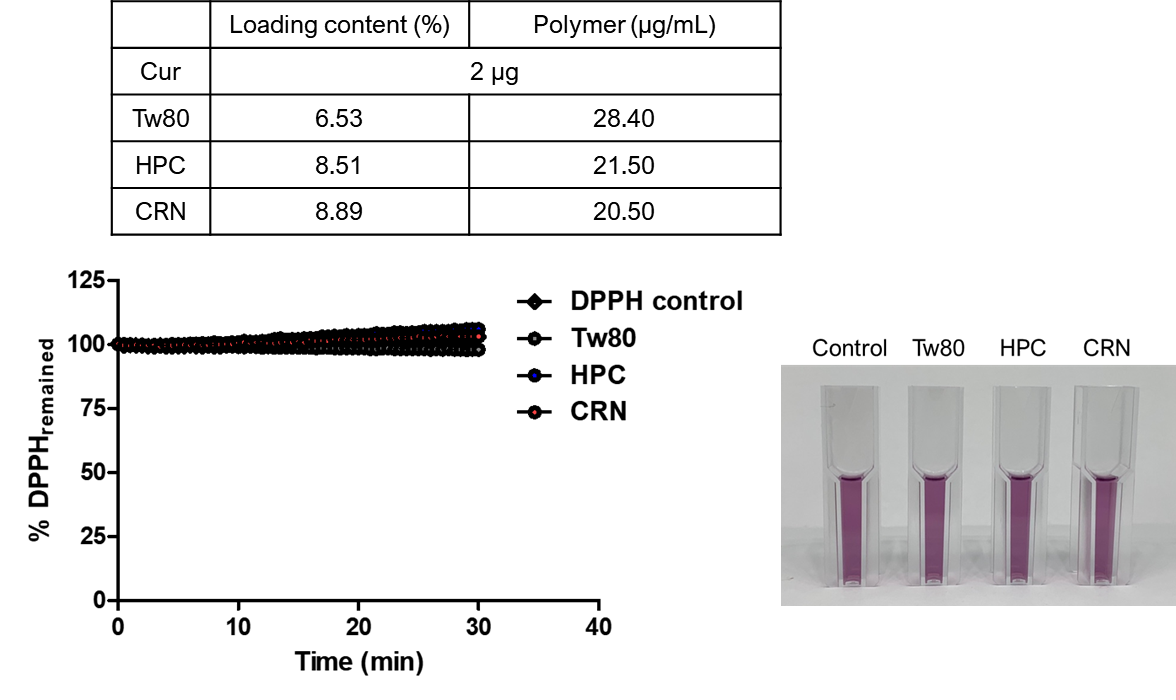


**Fig. S3.** DPPH bleaching kinetics in the presence of free Tw80, HPC, and CRN at 515nm for 30 min. The top table indicates the amount of polymer occupied per 2µg of Cur based on the converted loading contents (Table 1). Image of colorimetric change form purple to yellow.


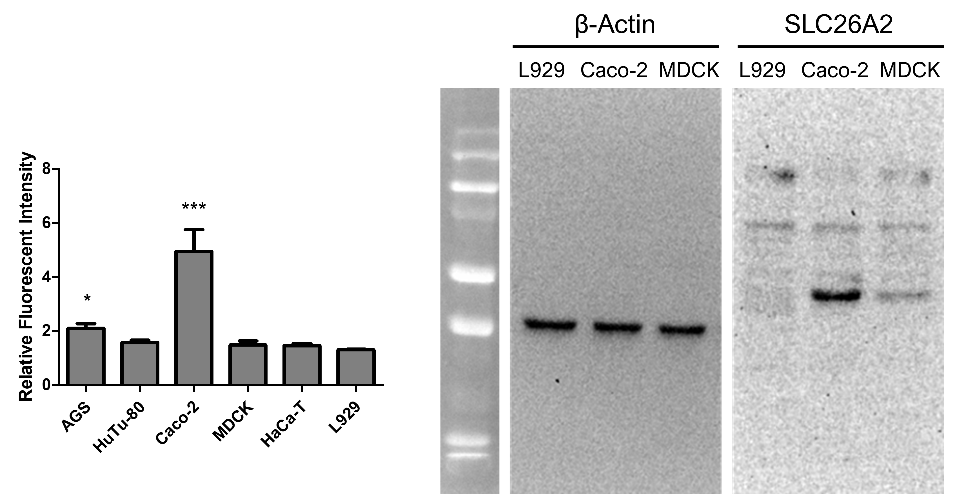


**Fig. S4.** Comparison of the expression level of SLC26A2 in L929, Caco-2, and MDCK cells (Positive control; Caco-2, Negative control; L929 and MDCK).

**MDCK**

| **Compounds** | **Direction** | **P_aap_ (×10^-6^ cm/sec)** | **Efflux ratio**  **(B to A/A to B)** |
| --- | --- | --- | --- |
| **Cur** | A to B | 0.0185 ± 0.0021 | 0.0809 |
|  | B to A | 0.0014 ± 0.0004 |  |
| **Cur@CRN** | A to B | 0.8228 ± 0.0620 | 0.0059 |
|  | B to A | 0.0048 ± 0.0015 |  |
| **Cur@Tw80** | A to B | 0.4294 ± 0.0470 | 0.0356 |
|  | B to A | 0.0153 ± 0.0005 |  |
| **Cur@HPC** | A to B | 0.6262 ± 0.0166 | 0.0034 |
|  | B to A | 0.0021 ± 0.0011 |  |

**Caco-2**

| **Compounds** | **Direction** | **P_aap_ (×10^-6^ cm/sec)** | **Efflux ratio**  **(B to A/A to B)** |
| --- | --- | --- | --- |
| **Cur** | A to B | 0.7567 ± 0.0142 | 1.7803 |
|  | B to A | 1.3473 ± 0.1619 |  |
| **Cur@CRN** | A to B | 5.7003 ± 0.1402 | N.A. |
|  | B to A | N.A. |  |
| **Cur@Tw80** | A to B | 4.2430 ± 0.1323 | 0.0445 |
|  | B to A | 0.1887 ± 0.0095 |  |
| **Cur@HPC** | A to B | 2.8353 ± 0.5779 | 0.0700 |
|  | B to A | 0.1985 ± 0.0101 |  |

**Table S3.** Permeability of various compounds across the MDCK and Caco-2 cell monolayers (n=3).


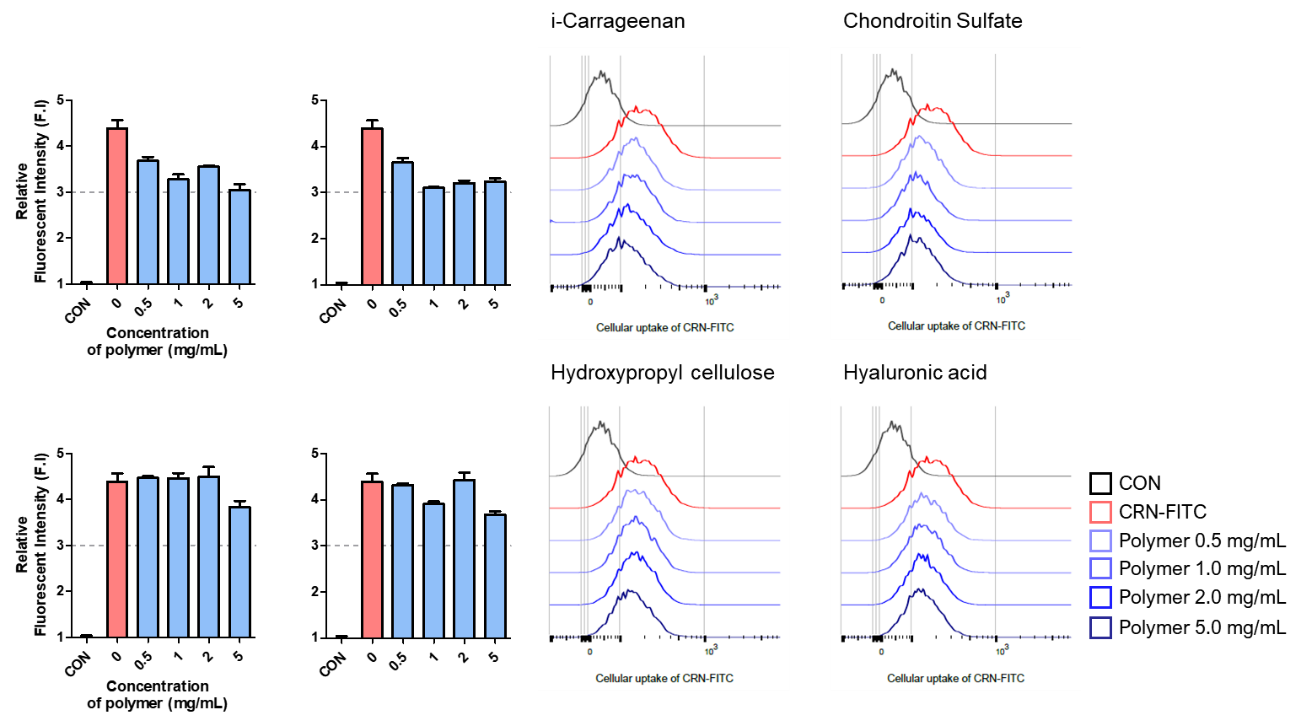


**Fig. S5.** Competitive inhibition test by co-incubating FITC-labelled CRN with various polymers in Caco-2 cells (n=3).
